# Mr. Spencer, on Hydrocephalus

**Published:** 1810-04

**Authors:** Samuel Spencer

**Affiliations:** Duffield


### *To the Editors of the Medical and Physical Journal.*

GENTLEMEN,

I Did hope that some more able pen would ere now have been employed in marking what I consider the inconsistencies and erroneous notions in some of Dr. Roberton's late papers, which, though trifling perhaps in themselves, are remarkable in coming from one of his assumed acumen, and who is so often adverting, with no little severity, to the faults, or supposed inaccuracies and misapprehensions of others. I shall confine myself in these remarks more particularly to his paper on hydrocephalus, which appeared in one of your late Numbers. He is pleased there, very erroneously I hope, to call this the disease of children, "now," as he gravely asserts, "that the small-pox has been driven out of this Country."—The small-pox driven out of this Country!—Here we have one of those loose and sweeping assertions, so frequently occurring in the Doctor's papers. Again, "that as many, if not more, die of it than even died of the small-pox," is another of his bold assertions, which every practitioner will know how to appreciate.

Doctor R. entitles his paper "Original Remarks on the Pathological Theory and Practical Treatment of Hydrocephalus Internus, illustrated by cases, &c." So conspicuous a title was well calculated to catch the attention of your readers, and I confess I was one of those who entered upon it with great pleasure, hoping that it might lead to a surer discrimination and more successful practice in that too often fatal malady. But seldom, surely, was attention more more ill bestowed, or expectation more severely disappointed. For after passing through an introduction, to me at least most truly unimportant, we are led on to a detail of cases, which, generally speaking, are clearly referable to another source of disease; and instead of being illustrative of the disease in question, are calculated only to confound it with other infantile ailments.

According to the "Pathological Theory" of Dr. Roberton, we are, on every occurrence of fever in children, with apparent head-ach, torpid bowels, or dark coloured stools, to act under the idea of the presence of hydrocephalus; and without further consideration, doom the little sufferer to a severity of treatment which its case, perhaps, by no means required. For how often do we meet with cases, such as he has described to us, giving way to active purgation alone? From whence we conclude, that the head, if at all, was not primarily affected; and that the "cap blister" might oftentimes have been well dispensed with. The doctor in his remarks, proves to us, that it is an easy matter to make a confident assertion; but to be able to discern and to paint to the view of others, "the distinctive physiognomy of diseases, the sort of shade between sameness and difference in symptoms," is another consideration, which he does not care seemingly to involve himself with. "He considers all the diseases of children to be hydrocephalus," as Dr. Fogo has well observed.

Dr. Roberton, as if to complete his subject, goes on with it in the two succeeding Journals, in his "Reports of the Diseases of Edinburgh." At length, he thinks he has said enough, and then sums up by asserting that he has given us "a new view of this disease, and a very effectual mode of subduing it." Surely Dr. Roberton cannot be dealing seriously with us. As to his new view, we have already examined it, and his effectual mode of subduing this disease, when it really exists, unfortunately consists only in that sort of treatment which has disappointed many an anxious practitioner before him. To conclude. We cannot suppose Dr. Roberton ignorant of the best Treatises which we have on this malady; and yet, with these in his recollection, it is hard to think what could induce him to publish his "Original Remarks;" or on what ground he could hazard so confident a proposition as that just quoted. Does he court a comparison with any of the best writers who have preceded him on this subject? His fame we think would be no ways enhanced by bringing before him the Essay of the learned Dr. Whytt, published on this subject subject more than half a century ago; the stile and manner of which is well worthy of imitation.

The appearance of your last Number induces me to make one further observation. Dr. Roberton, I hope, before this paper meets his notice, will have read the excellent "remarks on the infantile fever," by Junius, in this number; and if he then persists in thinking that his own cases characterise hydrocephalus, we would recommend to him the reconsideration of the last sentence in his "Reports of the diseases in Edinburgh, for December 1809," where he speaks of "mulish stubbornness," and "Scotish obstinacy." With what justice the Doctor applies these coarse epithets to his Countrymen, we will leave to the consideration of those who are better able to determine. True it is, that in himself, we have not that full manifestation of mental pliancy which might have been expected in one so suddenly sprung up from "the hot-bed of genius." But the "soil," we fancy, wants ameliorating, it being at present, as he tells us, "much calculated for the production of obstinacy." It may be so; but we have no proof of it in the writings or acknowledged conduct of those, who first and most adorned the great "Medical School" of Edinburgh; in the bosom of which, probably, the Doctor was nurtured; but he failed to imbibe, seemingly, that spirit of meekness, which the introduction to, and pursuance of our profession is so well calculated to inspire.

### *Observations on Mr.* Davies's *Paper, on Uterine Hæmorrhage.*

The "cautionary hints," or rather practical dogmas, of your respectable correspondent Mr. Davies, on uterine hæmorrhage, are better calculated, in my opinion, to give strength and interest to the minds of the experienced, than "to prove useful," as he hopes they may, "to the junior practitioners in midwifery." The minds of young men are not to be formed, nor can their rules of practice be taken from such confined limits as Mr. Davies has assumed to himself in his paper. He did not, I dare say, think that his "Observations" should at all supersede more enlarged reading; but recommended as they are to the notice of the junior practitioners in particular, they inculcate, I think, a position or two of a dangerous tendency. To illustrate. "In other cases of difficulty," says Mr. Davies, "the practitioner has leisure to deliberate on the best mode of relieving his patient, without risking any conseconsequences." As if to proceed hastily and indeliberately in this instance, were not risking the most serious consequences. I should advise the young practitioner, even in these cases, not to decide too hastily, or be afraid of deliberating on the best mode of relieving his patient. Let him, if he has an opportunity, call a consultation, rather than hazard his reputation in a too early attempt to deliver, which it is allowed, "in some cases will be attended with manifest discouragement." Let him not be persuaded to admit it as a general unqualified rule, that, "the sooner an attempt is made to deliver, the greater probability there will be of saving his patient." Cases, no doubt, do and will occur, where "the violence of the symptoms demand immediate help," where delivery must be proceeded in without waiting for pains, or for any considerable dilatation of the os uteri. It is hardly possible for the most able to lay down rules that will adapt themselves to every variety of case that may fall out in practice. It may be argued on the one hand, that "delays are dangerous," and on the other, that "proper delays will be advantageous."

But, after all, every thing must depend upon the individual view of the attending practitioner; though, it is a good general maxim, "to take as much time as symptoms will permit." A strict examination as to the attachment of the placenta, and a mind well stored with the facts and observations contained in Dr. Rigby's excellent pamphlet on "Uterine Hæmorrhage," will best serve a young man in these anxious cases.

As to the "deplorable case" to which Mr. Davies alludes, I know nothing; and with that gentleman, most sincerely do I hope I never may. It seems, however, that such cases have occurred in practice, and are acknowledged by the "best authorities," and it is well therefore that practitioners should be apprized of the chance of meeting with them.

Is it a fact, as we have long been accustomed to consider it, that in cases of placenta presentation, the hæmorrhage uniformly increases in proportion to the dilatation of the os uteri, and that nothing consequently is so much to be dreaded as the natural progress of the labour? If so, whence is it, that we hear of cases of hæmorrhage from the same presentation proving suddenly fatal, before the os uteri shews any disposition to dilate, or while it is "incapable of any dilatation whatever?"

I am, &c.

